# Generalised Sandpile Dynamics on Artificial and Real-World Directed Networks

**DOI:** 10.1371/journal.pone.0142685

**Published:** 2015-11-25

**Authors:** Nicky Zachariou, Paul Expert, Misako Takayasu, Kim Christensen

**Affiliations:** 1 Department of Physics, Blackett Laboratory, Imperial College London, Prince Consort Road, South Kensington Campus, London SW7 2AZ, United Kingdom; 2 Centre for Complexity Science, Electrical & Electronic Engineering Building, Imperial College London, South Kensington Campus, London SW7 2AZ, United Kingdom; 3 Centre for Neuroimaging Sciences, Institute of Psychiatry, Psychology and Neuroscience, De Crespigny Park, King’s College London, London SE5 8AF, United Kingdom; 4 Department of Computational Intelligence and Systems Science, Interdisciplinary Graduate School of Science and Engineering, Tokyo Institute of Technology, 4259 Nagatsuta-cho, Midori-ku, Yokohama 226-8502, Japan; Nankai University, CHINA

## Abstract

The main finding of this paper is a novel avalanche-size exponent *τ* ≈ 1.87 when the generalised sandpile dynamics evolves on the real-world Japanese inter-firm network. The topology of this network is non-layered and directed, displaying the typical bow tie structure found in real-world directed networks, with cycles and triangles. We show that one can move from a strictly layered regular lattice to a more fluid structure of the inter-firm network in a few simple steps. Relaxing the regular lattice structure by introducing an interlayer distribution for the interactions, forces the scaling exponent of the avalanche-size probability density function *τ* out of the two-dimensional directed sandpile universality class *τ* = 4/3, into the mean field universality class *τ* = 3/2. Numerical investigation shows that these two classes are the only that exist on the directed sandpile, regardless of the underlying topology, as long as it is strictly layered. Randomly adding a small proportion of links connecting non adjacent layers in an otherwise layered network takes the system out of the mean field regime to produce non-trivial avalanche-size probability density function. Although these do not display proper scaling, they closely reproduce the behaviour observed on the Japanese inter-firm network.

## Introduction

A long standing problem in macroeconomic theory is explaining the large fluctuations in aggregate economic activity that result from many small, independent shocks to individual sectors. Economists are particularly interested in understanding the observed instability that a constant demand can have to the economic aggregate, since this is very costly to inventory management and scheduling at the production level [1]. Understanding the mechanisms hidden behind the behaviour of the economy as a whole, which lead to these large scale fluctuations, is not easy. Naturally, one would expect that the demand and production in different parts of the economy would be independent and hence independent shocks to different sectors would cancel in the aggregate leading to Gaussian distributed fluctuations. Unfortunately, this is not the case, which leads to the speculation that there are significant non linear and strongly localised interactions between the seemingly independent sections of the economy. A paradigm that has been proposed to explain the fluctuations on all scales caused by small independent shocks is self-organised criticality [1, 2].

Consider a pile of sand. Dropping constantly one grain of sand at the time on top of this pile, will eventually trigger an avalanche, where some grains will topple down the slope. The size of the avalanche is defined as the number of grains involved in the toppling and interestingly, after a transient period, the avalanche-size probability density function (pdf) can be scale invariant [3]. Behaviour such as this is not only observed in the aggregate economic activity [1], but also in nature [4, 5], from lung inflation [6] and coevolution of species [7] to earthquakes [8, 9] and rainfall [10]. Self-organised criticality, a term coined by Bak, Tang, and Wiesenfeld (BTW) refers to systems with many degrees of freedom that spontaneously reach a dynamically critical state when slowly driven [3]. Once the critical state is reached, the self-organised critical system relaxes by a burst of activity. The avalanche sizes *y* display finite-size scaling (FSS), that is, follow a power-law pdf, $P\left(y;L\right)=y^{-\tau }G\left(y/L^{D}\right)$ for *y* ≫ 1 and *L* ≫ 1, where the critical exponents *τ* and *D* are the so-called avalanche-size exponent and the avalanche dimension, respectively, and G is a universal scaling function [11, 12]. This property of self-organised critical systems is induced by the long-range spatial and temporal correlations that exist, via short range interactions only, when the system has naturally evolved into the critical state. The directed sandpile model is a special case of the BTW model, where relaxation follows a directional rule [13], in the sense that there is a flow of motion which cannot be reversed. The dynamics is still critical, with scale invariant avalanche-size pdf obeying finite-size scaling. Dhar and Ramaswamy have shown analytically that such a directed sandpile model on a two-dimensional lattice has a power-law avalanche-size pdf with scaling exponents *τ* = 4/3 [13] that yields *D* = 3/2 [11].

Bak, Chen, Scheinkman and Woodford [2] showed that the classical two-dimensional directed sandpile dynamics can be used as a model for intersectoral trade and in their attempt to investigate the fluctuations in the production they recovered the same scaling exponents *τ* = 4/3 and *D* = 3/2. We adapted the dynamics of [2, 13] to cover for different number of producers and consumers and run the dynamics on less rigid topologies.

The most realistic topology one can use for this task is the production-consumption network between firms in a real economy, such as the Japanese inter-firm network. The Japanese inter-firm data set, is a snap shot of the entire production network of all active firms in Japan, for the year 2005, where firms are represented as nodes with directed edges showing the flow of orders (or money) from the customer node to the supplier node. In comparison with the regular lattice, this network is not rigid and has a scale-free distribution both in the in- and out-degrees. It is also a very shallow network with short average path length and has many triangles, cycles and motifs [14]. On the Japanese network, we find an avalanche-size exponent of approximately *τ* = 1.87. Although the system is still critical and reproduces qualitatively the behaviour observed by economists on aggregate production, this a novel avalanche-size exponent found which has not been observed before.

With the recent advance of complex networks theory, the behaviour of the BTW on non-lattice substrate have been investigated. In a series of papers, Goh *et al*. [15–18] have investigated the BTW model on scale-free undirected networks. Using a branching process approach, they found two regimes for the avalanche-size exponent *τ* as a function of the degree exponent *γ* of scale-free networks: *τ* = *γ*/(*γ* − 1) for 2 < *γ* < 3 with logarithmic corrections for *γ* = 3 and the classical mean field avalanche-size exponent *τ* = 3/2 which is recovered for *γ* > 3. The branching process is an inherent mean-field approach, as the cascading processes are uncorrelated, thus it is interesting to see that highly heterogenous networks, even treated with a mean-field approach yields an avalanche-size exponent different from the mean-field one. The behaviour of the BTW model has also been studied on undirected [19] and directed [20] small world network generated starting from a regular lattice with bidirectional links. While the system is in the mean-field universality class for all values of the rewiring probability *p* in the directed case, there is a cross-over from the BTW universality class when *p* = 0 to the mean-field universality class when *p* > 0 in the undirected case. Even though there have been studies of the sandpile model on complex networks, the case of directed sandpile has yet to be investigated. If one is to understand where this new avalanche-size exponent of *τ* = 1.87 comes from, then one must break down the transition of the directed sandpile from the layered lattice, whose behaviour is very well known and documented, to the directed sandpile on a real world network of the economy. So how are these networks different and what are the ingredients needed to go from one to the other?

Firstly, we must account for the distribution of the in- and out-degrees. Thus, we keep the strictly layered structure of the lattice but we introduce an interlayer degree distribution, such as a Gaussian or the more realistic truncated scale-free distribution. The moment we introduce any interlayer heterogeneity, we observe scale invariant avalanche-size pdfs obeying FSS with the mean field scaling exponents. Clearly, this modification is not enough.

Secondly, we must account for the shallowness of the network and introduce some cycles, triangles and motifs. To do so, we relax the strictly layered constraint by allowing a small proportion of long range connections across layers both in the direction of flow and opposite to it. This last perturbation is very similar in nature to the perturbation that Watts and Strogatz used in their seminal paper to modify a regular lattice and turn it into a small-world network [21]. Now, the avalanche-size pdfs show fat tails with an avalanche-size exponent close to the one observed in the Japanese interfirm network. However, with the introduction of random long-range connections, the number of layers *L* is no longer the characteristic scale of the system; it is therefore not possible to perform FSS of the avalanche-size pdfs.

The long range connections bring the system out of the mean field behaviour and produce non-trivial avalanche behaviour which can closely reproduce the one observed using the Japanese inter-firm network. We believe that we have identified perturbations to a strictly layered sandpile model that produces a non-trivial avalanche-size pdf consistent to what is observed on a real-world Japanese inter-firm network.

## Methods

### Model definition

Bak, Chen, Scheinkman and Woodford [2] showed that the classical two-dimensional directed sandpile dynamics can be recast in the language of intersectoral trade in their attempt to investigate the fluctuations in the production. They assumed that the economy has the form of a cylindrical lattice with *L* layers, where each productive unit is connected to two suppliers and two consumers directly in the layer below and above it, respectively. During production, two units of output are created by utilising two units of input taken equally from each supplier. To optimise the costs of keeping inventory, each unit can hold a maximum of one unit of new product. They independently shock the first layer of the economy and monitor the aggregate production at that period. Production occurs when an order from a customer cannot be fulfilled exclusively by utilising products from the suppliers inventory [1, 2]. The distribution of the aggregate production follows exactly the behaviour of the avalanches in the directed sandpile model described above, with the avalanche-size scaling exponents *τ* = 4/3 and *D* = 3/2. This simple model was successful in qualitatively recreating the desired behaviour observed in the macro-economy; however, it has many drawbacks, the biggest of all being the very rigid and unrealistic layered topology used with a constant number of suppliers and customers per unit [1].

We now adapt the dynamics introduced by Bak, Chen, Scheinkman and Woodford [2] in a directed two-dimensional lattice, with co-ordination number *K* = 2, to a general lattice structure. Consider an economy made of *N* productive units arranged in *L* number of layers on a lattice with periodic boundary conditions connecting the left edge to the right edge. The width of the economy is defined as the circumference *C* of the lattice, where *C* = *N*/*L*.

We identify each productive unit by its coordinates in this 2-dimensional system, which are denoted by (*i*, *j*). Each productive unit has a set of suppliers (below) to buy its supplies from and a set of customers (above) to sell its products to. In the layered economy this is denoted by an unweighted directed link (*n*, *n*′) going from each supplier node *n*(*i*, *j*) to each customer node *n*′(*i*′, *j*′). Thus, the set of customers of node *n*(*i*, *j*) is defined as *N*_*i*, *j*_ = {*n*′(*i*′, *j*′)∣*n*′(*i*′, *j*′) is a customer of *n*(*i*, *j*)}.

**Fig 1:**
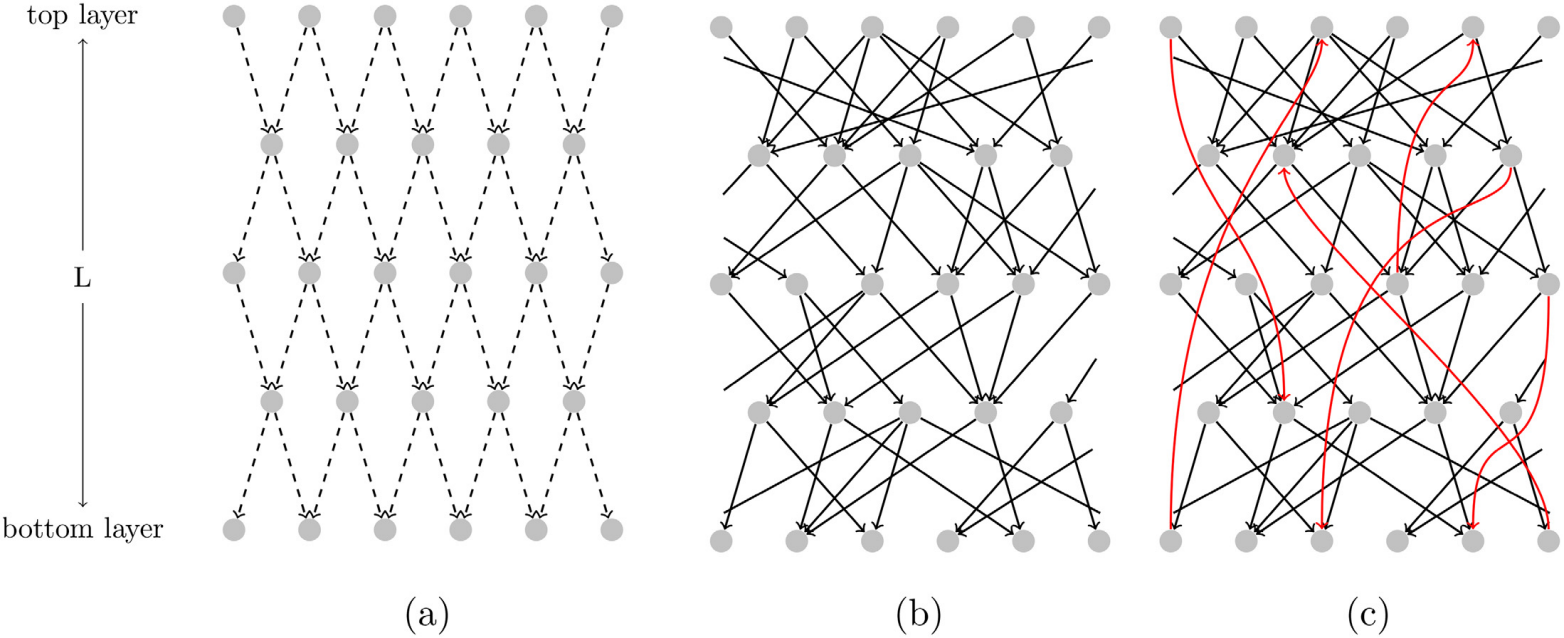
Diagram illustrating the different topologies on a subset of the network. (a) Dashed black lines: two-dimensional directed lattice with coordination number *K* = 2. (b) Black solid lines: layered network with a randomised degree distribution (e.g. Gaussian or Scale-free) with randomly chosen neighbours in the adjacent layer below. (c) Black solid lines and red solid lines: networks created by adding links connecting non adjacent layers (red) in both directions to the layered network with randomised degree distribution (black).

The underlying network of the economy can be drawn by accumulating all the links, where the in- and out-degrees of each node, $k_{i,j}^{in}$ and $k_{i,j}^{out}$, are defined as the number of customers and suppliers, respectively, each company *n*(*i*, *j*) has. In such a system, the top layer can be thought of as the final goods producers, the bottom layer as providers of primary inputs (raw materials) and anything in between as the intermediate goods producers. Fig 1 is an illustration of a subset of such a network.

At each time-step a production unit may be activated and asked to sell to its customers. In this case, the variables of interest for company *n*(*i*, *j*) are *x*_*i*, *j*_(*t*): the state of the inventory at time *t*, *y*_*i*, *j*_(*t*): the quantity of goods produced at time *t* and *s*_*i*, *j*_(*t*): the number of goods sold at time *t*. When asked to sell, the productive unit will check its inventory to see if there is enough to sell without having to produce. If this is the case, then *y*_*i*, *j*_(*t*) = 0, otherwise it produces enough to cover for all its customers, $y_{i,j}\left(t\right)=k_{i,j}^{in}$. Based on these genaralised dynamics, the variables of interest can take the following values, which are dependent on the in- and out-degrees of each individual company node: $x_{i,j}\left(t\right)\in \left\{0,\ldots ,k_{i,j}^{in}-1\right\}$ for the inventory size; $y_{i,j}\left(t\right)\in \left\{0,k_{i,j}^{in}\right\}$ for the production and $s_{i,j}\left(t\right)\in \left\{0,\ldots ,k_{i,j}^{in}\right\}$ for the sales. We also impose that $k_{i,j}^{in},k_{i,j}^{out}\geq 2$ to avoid dissipation.

Production is induced when a customer’s order cannot be fulfilled exclusively by utilising products from the inventories of its suppliers. The inventory of each activated production unit is updated from its previous state by adding the number of units it produced at that time-step and subtracting the number of units it sold. Hence, the equations that govern the inventory dynamics can be cast in the following general form:

$$x_{i,j}\left(t+1\right)=x_{i,j}\left(t\right)+y_{i,j}\left(t\right)-s_{i,j}\left(t\right),$$ (1)

where the orders received by each unit are accumulated over all its active customers at that time-step

$$s_{i,j}\left(t\right)=\sum_{n^{\prime }\left(i^{\prime },j^{\prime }\right)\in N_{ij}}\frac{y_{i^{\prime },j^{\prime }}\left(t\right)}{k_{i^{\prime },j^{\prime }}^{in}}.$$ (2)

Thus, an avalanche is defined as the total size of production, *y*(*t*), and is denoted by the sum of the productions over all *n* = 1, …, *N* nodes in the system.

$$y\left(t\right)=\sum_{n=1}^{N}y_{i,j}\left(t\right).$$ (3)

Only activated nodes will give a contribution at each time step. It is worth noting that irrespective of the amount of production of a certain node, it will give its customer one unit of raw materials. Reversely, regardless of how much raw materials a customer needs it can ask for one unit only from each of its suppliers.

### Theoretical framework

Even though a given avalanche size cannot be predicted unless one has complete knowledge of the whole system, the statistics of avalanche-size pdfs follow well-defined laws [11]. Assume that the avalanche-size pdf obeys simple FSS, that is,

$$P\left(y;L\right)=ay^{-\tau }G\left(y/L^{D}\right)\;\text{for}\;y\gg 1,L\gg 1,$$ (4)

where *τ* and *D* are universal avalanche-size scaling exponents, G a universal scaling function and *a* a non-universal dimensionful parameter independent of lattice size L. If data for different system sizes *L* are consistent with the FSS ansatz in Eq (4), then we can perform a data collapse because simple manipulation of Eq (4) yields

$$y^{\tau }\;P\left(y;L\right)=aG\left(y/L^{D}\right).$$ (5)

Hence, by plotting the transformed probability density function, *y* ^*τ*^ *P*(*y*;*L*) versus the rescaled observable *y*/*L* ^*D*^, all the data collapses onto the curve for the scaling function G.

We can estimate the avalanche-size scaling exponents using moment analysis. Assuming that Eq (4) is valid for all *y*, and approximating the sum with an integral, we find that the *k*th moment

$$\begin{array}{ccc} \langle y^{k}\rangle \; & = & \;\sum_{y=1}^{\infty }\;y^{k}\;P\left(y;L\right)\;\propto \;\int_{1}^{\infty }\;\;\;y^{k-\tau }G\left(y/L^{D}\right)\;dy\propto \;L^{D(1+k-\tau )}, \end{array}$$ (6)

for *L* ≫ 1, *k* ≥ *τ* − 1. Plotting the directly measured *k*th moments 〈*y* ^*k*^〉 versus system size *L* in a double-logarithmic plot yields a straight line with slope *D*(1 + *k* − *τ*) for *L* ≫ 1. Plotting the estimated slopes *D*(1 + *k* − *τ*) versus *k* yields a straight line with slope *D* intersecting the *k*-axis at *k* ^⋆^ = *τ* − 1, that is, *τ* = 1 + *k* ^⋆^. We will use the moment scaling analysis to estimate *D* and *τ* and then check whether the estimated avalanche-size scaling exponents produce a data collapse according to Eq (5). Due to conservation we expect the first moment 〈*y*〉 ∝ *L* ^*D*(2−*τ*)^ ∝ *L*, that is *D*(2 − *τ*) = 1.

## Results

In the simulations presented below we used the Japanese inter-firm network (*N* ≈ 10^6^) as given to us, and/or used three lattice sizes *L* = 200, 400, 600, but kept the width of the system constant, *C* = 2000 where appropriate (*N* = 4⋅10^5^, 8⋅10^5^, 1.2⋅10^6^). We let the simulations run for a transient period to eliminate initialisation bias and bring the system into a steady state. The transient period we used for the inter-firm network was 10^6^ events, for the regular lattices the transient was 5 × 10^5^ avalanches and for the other networks the transient was 10^6^ avalanches. More data was needed as the randomness in the structure of the network makes it slower to reach stationarity. Then, we started recording the avalanches for an additional 10^7^ events on the inter-firm network and 10^6^ for all others. For the non-regular networks, we repeated the experiment on 5 different realisations of the networks and then averaged over all 5 experiments. The inventory of the first layer was kept empty to ensure that each event was non-zero.

To analyse the results we obtained from the simulations we calculated the *k*th moment directly. For the visualisation of the avalanche size pdfs, we used a logarithmic data binning method. We divide the horizontal axis into bins labelled *j* = 0, 1, …, where the *j*th bin covers the interval $[a^{j},a^{j+1}[\;=\left\{r\in R|a^{j}\leq r<a^{j+1}\right\}$. With *a* > 1, the bins are exponentially increasing in length. We let $y_{min}^{j}$ and $y_{max}^{j}$ denote the minimum and maximum integer avalanche sizes in bin *j*, and then count the number of avalanches that fall in bin *j*, with interval $\left[y_{min}^{j},y_{max}^{j}\right]$. We then plot *P*(*y*; *L*) against the geometric mean, $y^{j}=\sqrt{y_{max}^{j}y_{min}^{j}}$ of the avalanche sizes in bin *j* as defined below:

$$P\left(y^{j};L\right)=\frac{No.\;of\;avalanches\;in\;bin\;j}{N\Delta y^{j}}$$ (7)

where $\Delta y^{j}=y_{max}^{j}-y_{min}^{j}+1$ is the number of integers in the interval $\left[y_{min}^{j},y_{max}^{j}\right]$ and *N* the number of avalanches. The logarithmic binning we use, with *a* = 1.1 allows us to extract information on *P*(*y*) which would have been impossible to see in the noisy tail of our pdf graphs [12].

In the remainder of this section, we present the simulation results organised as follows: in Sec. A we show the behaviour of the generalised BTW dynamics when run on the Japanese inter-firm network; in Sec. B we show the results from the regular lattice and recover the theoretical results obtained in [13] to check our numerical framework; in Sec. C we relax the in- and out-degree distributions constraint but keep the strictly layered nature of the lattice and finally in Sec. D we relax the layeredness constraint.

### Japanese inter-firm network

The generalised BTW sandpile dynamics, can be used to model the production network of a country; it is analogous to our simple view of production, but without the rigidity of the strictly layered system. Instead there is a web of production with directed links imposing a direction of flow which is not strictly layered.

The Japanese inter-firm data set, which was collected by Tokyo Shoko Research Ltd. (TSR) and provided by Research Institute of Economy, Trade and Industry (RIETI), is a snap shot for the year 2005 of the entire production network of all active firms in Japan. It represents the firms of Japan as nodes joined with directed links showing the money flow from the customer node to the supplier node. With the analogy between the directed sandpile and the inter-firm trade we made, it is pertinent to study how such a simple model behaves on the production network of an entire country.

In this network there are around 1 million nodes and 4 million links. Previous analysis of the structure of the network has shown that the in- and out- degrees distributions are scale-free with exponents *γ* = 2.35 and *γ* = 2.26, respectively. Moreover, the average path length in the network is 5.62 and the maximal distance is 21 making it a very shallow network. The network has a bow-tie structure, in the sense that there is a distinct in-component where 31% of nodes have $k_{i,j}^{in}=0$, a strongly connected component comprised of 53% of the nodes and a strict out-component formed by the remaining nodes with $k_{i,j}^{out}=0$ [14]. We will treat the in-component as the top layer and interpret it as the final retailers where all propagation begins, the out-component as the bottom layer formed of primary producers where final orders are placed and observe how avalanches of production fluctuate within the strongly connected component. The constraints on the inventory, production and sales of each firm remain the same and are defined by the in-degree of each firm.

**Fig 2:**
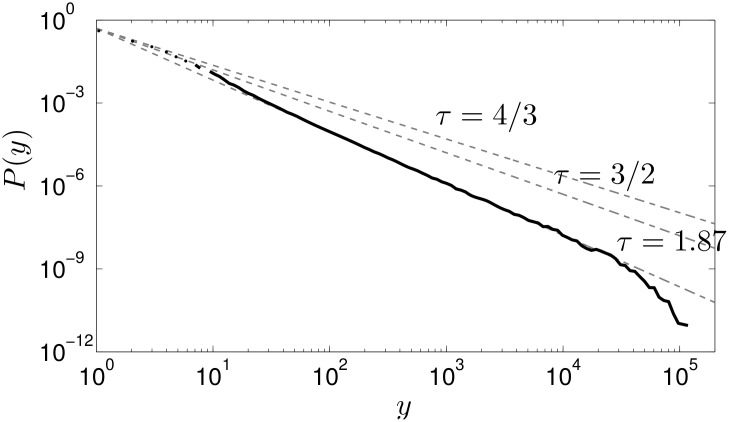
The avalanche-size pdf *P*(*y*) versus the avalanche size *y* obtained using the inter-firm Japanese network (solid black line). The grey dashed lines are guides to the eyes for the different universality classes’ avalanche-size exponents.

The avalanche-size pdf, which is shown in Fig 2 (solid black line), display a broad distribution that is consistent with a power-law decay over three orders of magnitude, with an estimated avalanche-size exponent *τ* = 1.87. This clearly different from the two universality classes characterised by avalanches size exponents *τ* = 4/3 and *τ* = 3/2 (grey dashed lines).

### Regular lattice

**Fig 3:**
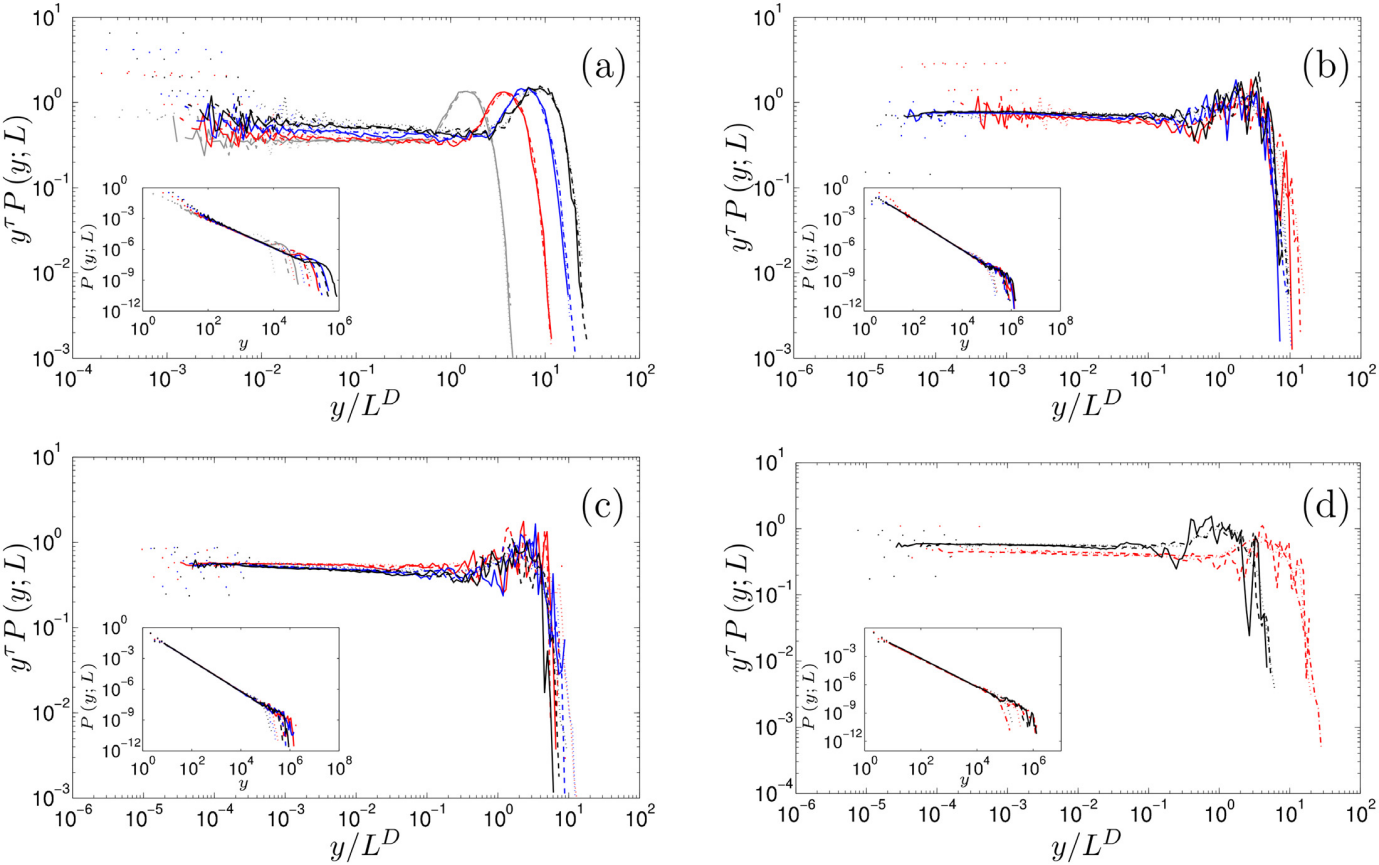
For all panels, the inset displays the avalanche-size pdf *P* (*y*; *L*) vs. the avalanche size *y*. The large figures show the data collapse obtained by plotting the transformed avalanche-size pdf *y* ^τ^ *P* (*y*; *L*) vs. the rescaled avalanche size *y*/*L* ^*D*^ using the estimates of the avalanche-size scaling exponents τ and *D* obtained from moment scaling analysis, see Tables 1, 2, 3 and 4. For all figures, including insets, the line style indicates the system size, dashed-dotted: L = 100; dotted line: L = 200; dashed line: L = 400; solid line: L = 600 (a) Regular lattice; grey: K = 2, red: K = 4, blue: K = 6, black: K = 8, *L* = 200, 400, 600 (b) Gaussian out-degree distribution; red: σ = 0, blue: σ = 1, black: σ = 2, *L* = 200, 400, 600 (c) Truncated scale-free out-degree distribution; red: γ = 2.5, blue: = 3.0, black: = 3.5, *L* = 200, 400, 600 (d) Truncated scale-free in- and out-degree distribution; red: γ = 2.5, *L* = 100, 200, 400, black: γ = 3.5, *L* = 200, 400, 600.

First, we reproduce the result of Bak *et al*. [2] on the regular lattice with fixed coordination number K = 2. We then go one step further by increasing the coordination number and observe the change this has on the avalanches produced. In this extension of the original model, we have that $k_{i,j}^{in}=k_{i,j}^{out}=K$, where $K\in N^{+}$ and *K* ≥ 2. Following the dynamical rules as prescribed in the Model definition section, we have the following constraints on the range of inventory *x*_*i*, *j*_(*t*) ∈ {0, …, *K* − 1}, production *y*_*i*, *j*_(*t*) ∈ {0, *K*} and sales *s*_*i*, *j*_(*t*) ∈ {0, …, *K*} for all nodes in the system. All exponents are consistent with the universality class of the two-dimensional directed sandpile model. The results are displayed in Fig 3(a) and the associated scaling exponents estimated using the moment analysis method are reported in Table 1. For each system, both scaling exponents are within error bars of the universality class of the two-dimensional directed sandpile model. The pdfs display the characteristic “bump” in their tails. This is a typical finite-size effect and is due to the fact that the number of avalanches that reach the last row is not only avalanches of that size but also all the other avalanches that would have carried on.

**Table 1:** The avalanche-size exponent, *τ*, and the avalanche-dimension, *D*, for regular lattice structures with coordination numbers, *K* = 2, 4, 6, 8 and circumference *C*, see Fig 3(a) for the data collapse. The scaling relation *D*(2 − *τ*) = 1 is fulfilled and, within error bars, both scaling exponents (apart from *K* = 8 and *C* = 2000) are consistent with the universality class of the two-dimensional directed sandpile model *τ* = 4/3 and *D* = 3/2. The numerical result for *K* = 8, *C* = 2000 and 4000 suggests that the apparent drift is due to finite size effects.

K	C	*τ*	*D*	*D*(2 − *τ*)
2	2000	1.33(5)	1.47(10)	0.98
4	2000	1.35(5)	1.55(10)	1.00
6	2000	1.37(5)	1.60(10)	1.00
8	2000	1.39(5)	1.63(10)	1.00
8	4000	1.37(5)	1.61(10)	1.00

We observe that *τ* and *D* seem to increase slightly as the coordination number *K* increases for the rigid networks (see Table 1). Comparing numerical results for *K* = 8 and *C* = 2000, 4000 suggests that the apparent drift is due to finite-size effects. Hence, we deduce that the model on a regular lattice falls within the classical universality class of *τ* = 4/3 and *D* = 3/2 for all K.

### Layered lattice with inter-layer distribution of interactions

A natural generalisation from the regular networks described above is to maintain the strictly layered structure of the lattice, but introduce heterogeneity in the in- and out-degree distributions of the production units, also referred to as nodes. The constraints on the range of the dynamical variables follow the rules as described in Model definition section, which are now node-dependent. With these in place we can proceed with our investigation into the different inter-layer distributions.

In the first setting, the out-degree of a node is drawn from a Gaussian distribution $k_{i,j}^{out}∼N\left(\mu ,\sigma^{2}\right)$ and its neighbours chosen at random from the layer below it avoiding multiple links between nodes. We rejected out-degrees smaller than 2. The central limit theorem ensures that the in-degree distribution will asymptotically follow the same Gaussian distribution as the out-degree. We have set the mean to *μ* = 4 and used three values for the standard deviation *σ* = 0, 1, 2. Note that when *σ* = 0, we obtain a rewired version of the regular lattice with *K* = 4. The results are displayed in Fig 3(b) and the associated scaling exponents estimated using the moment analysis are reported in Table 2. For each system, both scaling exponents are within error bars of the universality class of the mean-field model.

**Table 2:** The avalanche-size exponent, *τ*, and the avalanche-dimension, *D*, for networks with nodes’ out-degrees drawn from a Gausssian distribution with a fixed mean coordination number *μ* = 4, and standard deviations *σ* = 0, 1, 2. Note that the case of *σ* = 0 is just a randomly rewired version of the regular lattice with coordination number *K* = 4, see Fig 3(b) for the data collapse. Within error bars, both scaling exponents are consistent with the mean-field model *τ* = 3/2 and *D* = 2.

*σ*	*τ*	*D*	*D*(2 − *τ*)
0	1.45(5)	1.8(2)	1.00
1	1.47(5)	1.9(2)	1.00
2	1.48(5)	1.9(2)	1.00

In the second case, we draw the out-degree of each node from a scale-free distribution $k_{i,j}^{out}\propto K^{-\gamma }$ for *γ* = 2.5, 3.0, 3.5 and truncated at max(*k* ^*out*^) = 20. Links are then placed randomly between nodes in adjacent layers, avoiding multiple links between nodes. As in the previous case, the central limit theorem yields that the in-degree distribution is Gaussian. The results are displayed in Fig 3(c) and the associated scaling exponents estimated using the moment analysis are reported in Table 3. For each system, both scaling exponents are within error bars of the universality class of the mean-field model.

**Table 3:** The avalanche-size exponent, *τ*, and the avalanche-dimension, *D*, for networks with nodes’ out-degrees drawn from a truncated scale free distribution with exponent *γ* = 2.5, 3.0, 3.5, see Fig 3(c) for the data collapse. The central limit theorem ensures the distribution of in-degrees is Gaussian. Within error bars, both scaling exponents are consistent with the mean-field model *τ* = 3/2 and *D* = 2.

*γ*	*τ*	*D*	*D*(2 − *τ*)
2.5	1.48(5)	1.9(2)	1.00
3.0	1.47(5)	1.9(2)	1.00
3.5	1.46(5)	1.9(2)	1.00

In the last setting, the out-degrees of the nodes were drawn from a scale-free distribution $k_{i,j}^{out}\propto K^{-\gamma }$ for *γ* = 2.5, 3.5 and truncated at max(*k* ^*out*^) = 20. The in-degrees of the layer below were then drawn at random from the realised distribution of the layer above. The stubs are then matched avoiding multiple connections between nodes. The results are displayed in Fig 3(d) and the associated scaling exponents estimated using the moment analysis are reported in Table 4. For each system, both scaling exponents are within error bars of the mean-field model exponents.

**Table 4:** The avalanche-size exponent, *τ*, and the avalanche-dimension, *D*, for networks with nodes’ in- and out-degrees were both drawn from a truncated scale free distribution with exponents *γ* = 2.5, 3.5, see Fig 3(d) for the data collapse. Within error bars, both scaling exponents are consistent with the mean field-model *τ* = 3/2 and *D* = 2.

*γ*	*τ*	*D*	*D*(2 − *τ*)
2.5	1.50(5)	2.0(2)	1.01
3.5	1.48(5)	1.9(2)	1.00

Notice that for a degree distribution exponent of *γ* = 2.5 the branching process approach predicts an avalanche-size exponent *τ* = 5/3 [15] which differs from the exponent *τ* = 3/2 we find.

### Long range connections across layers

We have seen that any perturbation of the underlying network topology, which keeps the layered structure, puts the system into the mean field exponent *τ* = 3/2. The fact that the Japanese inter-firm network does not have a unique direction of flow in the strongly connected component, as it is not strictly layered, might be what gives rise to the avalanche-size exponent *τ* = 1.87. Indeed, the characteristic small average path length (5.62) of the Japanese inter-firm network is a direct consequence of the lack of unique direction of flow and the non strictly layered structure.

We proceed with this hypothesis and introduce a small proportion of long range interactions in an otherwise layered network, both in the direction of the flow and opposite to it. This modification mimics the shallowness of the Japanese inter-firm network by reducing the path length of the lattice and also creates cycles in the system. As in the case of the Japanese inter-firm network, the first layer plays the role of the in-component and the last layer the role of out-component. Unlike the Japanese inter-firm network, we do not impose having a strongly connected component in the middle.

**Fig 4:**
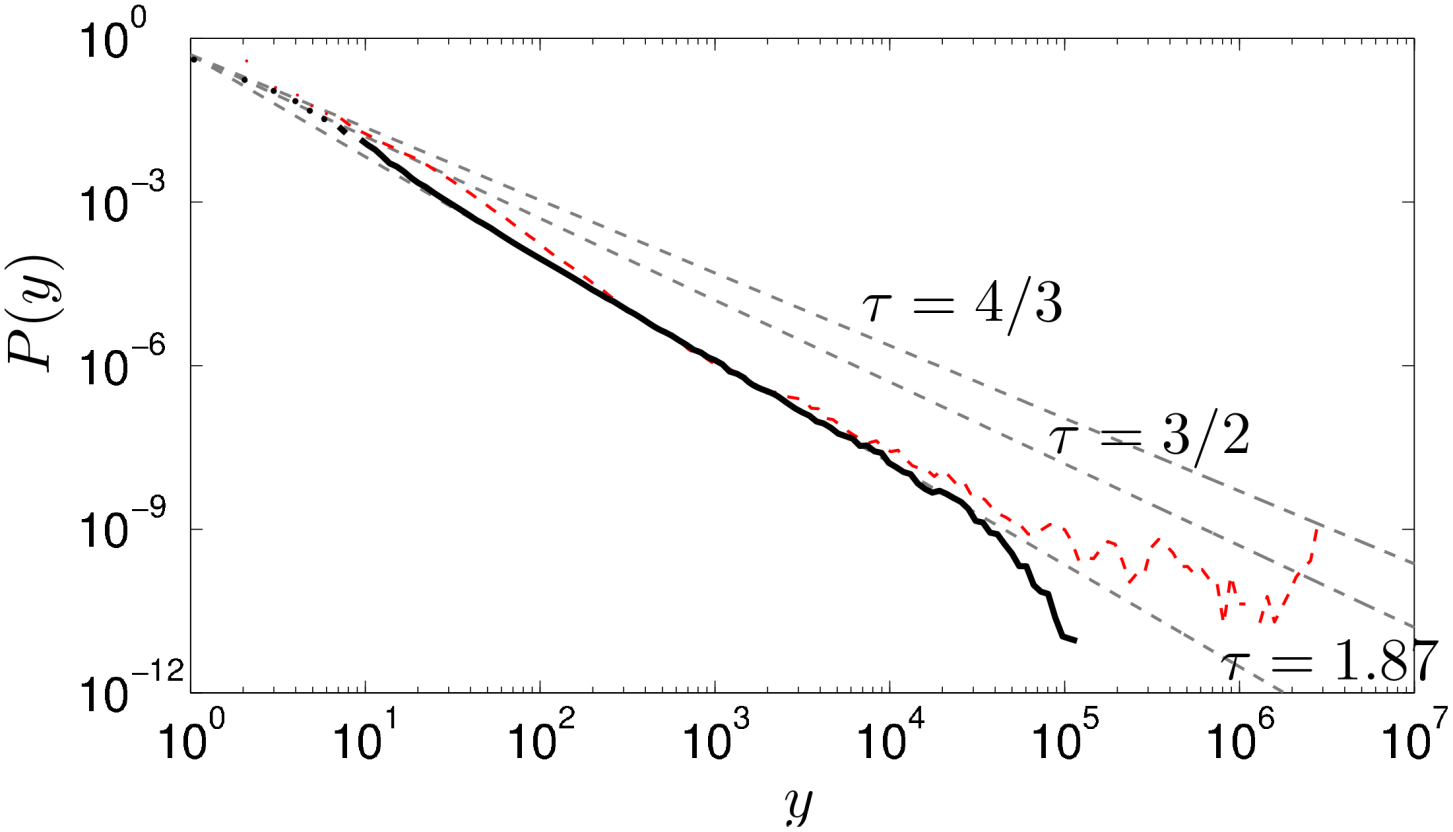
The avalanche-size pdf *P*(*y*) versus the avalanche-size *y* obtained using the inter-firm Japanese network (solid black line). With 25% of long range connections across layers in an otherwise layered network with nodes in- and out- degree drawn from a truncated scale-free distribution with exponent γ = 2.5 and system size *L* = 400 (dashed red line). The grey dashed lines are guides to the eyes for the different universality classes’ avalanche-size exponents.

We implement long range connections on the third type of networks described in the previous section, as they have scale-free degree distribution for both the in- and out- degree, like the Japanese inter-firm network. Each node has a probability *p*_*lr*_ to have one of its link connected to another random node in the network, with the only constraint that it is not in an adjacent layer. We ran simulations with *p*_*lr*_ ∈ [0.05, 0.10, 0.25, 0.50, 0.75]. This simple modification brings the system out of the mean field and produces a non-trivial avalanche behaviour. As shown in Fig 4, the case *p*_*lr*_ = 0.25 reproduces approximately the avalanche-size pdf found in the Japanese inter-firm network. For this particular network, the long range connections have reduced the average path length from 114 to 12, bringing it to the same order of magnitude as the Japanese inter-firm network which is 5.62. We want to point out that the real network did not evolve to its current structure from a layered structure, but rather evolved as a scale-free network that possess a bow-tie like structure with a strongly connected component. In a shallow strictly layered network, large avalanches are impossible. However, the introduction of long-range connections in such networks is an essential structural characteristic that allows the existence of large avalanches and hence a non-trivial avalanche-size pdf.

## Discussion

Characterisation of complex networks and measurements of them is still a developing field [22] that is relevant for a variety of research areas such as neuroscience [23, 24], finance [14] and computational chemistry [25–27] to mention a few.

Several conclusions and leads for future research can be drawn from our results. Many different universality classes exist for sandpile models depending on the dynamics considered, like the stochastic Manna model [28], whose universality classes have been studied in detail on directed lattices [29–32]. However, when considering the generalised deterministic BTW dynamics on strictly layered and directed lattices, and despite exploring the effect of in- and out-degree distribution in strictly layered networks, we only observe two universality classes: the classical two-dimensional directed sandpile model with *τ* = 4/3 and *D* = 3/2 and the mean field universality class with *τ* = 3/2 and *D* = 2 [13].

The intuition behind the existence of only two universality classes is that the highly constrained structure of strictly layered and regular lattices creates correlations in the activity, but these correlations are destroyed as soon as any rewiring is introduced that disturbs the rigid lattice structure, such as having the having the nodes connected in a random fashion between layers. For example, in the regular lattice where *K* = 4 we clearly observe the classical two-dimensional directed sandpile model class (see Table 1) but in the case where the out-degree of a node is drawn from a Gaussian distribution $k_{i,j}^{out}∼N\left(4,0\right)$ we clearly observe mean field behaviour (see Table 2). Since the latter example can be considered as a rewired version of the former, where nodes still have 4 neighbours but are connected randomly within the layers, we deduce that the transition between the two classes seems to be abrupt. For strictly layered lattice, we observed mean-field behaviour with Gaussian-Gaussian, Gaussian-Scale free and Scale free-Scale free in- and out- degree distributions.

However, when moving to a complex non-layered directed network, a new avalanche behaviour emerges with a non-trivial avalanche-size exponent of *τ* = 1.87. The avalanche-size exponent is steeper than both the two-dimensional directed sandpile model and the mean-field: 1.87 > 3/2 > 4/3. It is indeed rare to observe an avalanche-size exponent greater than the mean-field avalanche-size exponent *τ* = 3/2 in sandpile models [11].

We have shown that by simply adding a small proportion of random long-range links, and so destroying the strict layer structure of the system but without the constraint of having a strongly connected component, one can produce non-trivial avalanche-sizes pdf. Although these probability density functions are qualitatively close to the result obtained with the Japanese inter-firm network, no scaling is present, implying that other structural constraints need to be imposed. We cannot perform FSS because the structural heterogeneity induced by the presence of long-range links makes the effective system size (that would enter into FSS) not well defined.

We believe that these facts taken together may point towards the existence of new universality class for the directed sandpile model and that long-range links are an essential component. It would be of great scientific interest to determine whether such a new universality class indeed exists as the underlying structure giving rise to this behaviour is found in human engineered networks. We must also identify the finer structural properties that allow for SOC to appear, since the large events might be detrimental to the system. In the case of the economy, it is highly desirable if one could mitigate or even prevent these emerging large fluctuations in the production-consumption network.
